# Evaluating Hyperbolic Dispersion Materials for Cancer Detection

**DOI:** 10.3390/bios13060595

**Published:** 2023-05-30

**Authors:** Syed Muhammad Sohaib Zafar, Igor Iatsunskyi

**Affiliations:** NanoBioMedical Centre, Adam Mickiewicz University, 3 Wszechnicy Piastowskiej Str., PL-61614 Poznan, Poland; syed.zafar@amu.edu.pl

**Keywords:** label-free biosensor, hyperbolic metamaterials (HMM), cancer biomarkers

## Abstract

Current biosensors have limited application in clinical diagnostics as they lack the high order of specificity needed to detect low molecular analytes, especially in complex fluids (such as blood, urine, and saliva). In contrast, they are resistant to the suppression of non-specific binding. Hyperbolic metamaterials (HMMs) offer highly sought- after label-free detection and quantification techniques to circumvent sensitivity issues as low as 10^5^ M concentration in angular sensitivity. This review discusses design strategies in detail and compares nuances in conventional plasmonic techniques to create susceptible miniaturized point-of-care devices. A substantial portion of the review is devoted to developing low optical loss reconfigurable HMM devices for active cancer bioassay platforms. A future perspective of HMM-based biosensors for cancer biomarker detection is provided.

## 1. Introduction

Cancer treatment is a challenging and overwhelming task, prevention and detection can potentially save lives and reduce the overhead costs involved. According to recent statistics, one in every six cancer cases is fatal, and the global population of cancer patients has increased to ten million in 2020 alone [1].

**Scheme 1:**
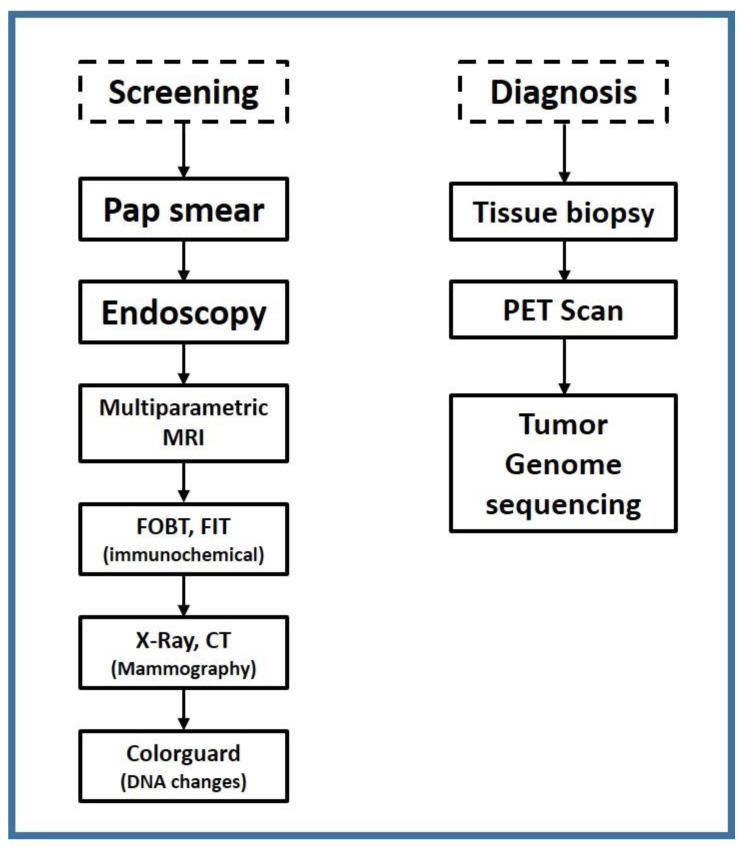
Cancer detection techniques [11].

A recent study by Prof. Carlo La Vecchia published in the 2022 issue of the Annals of Oncology reported that there were 1,269,200 cancer fatalities in Europe alone. Hurdles in early detection and prevention include cancer biology, evaluating the risk of developing cancer, validating cancer detection biomarkers, and technological development in early detection. Tumor models are required to understand transitions in cancer growth and development, for example, patient-derived tumor cell explants, organoids, and xenografts. Risk assessment graphs and population data sets with family history, lifestyle, and age groups will help predict genomic and phenotype biomarkers and genetic variants—for instance, women who inherit BRCA1 and BRCA2 are more susceptible to ovarian and breast cancer. Laboratory-based cancer screening techniques that are currently being implemented early are shown in Scheme 1 [1,2,3,4,5,6,7,8,9,10].

Early-stage diagnostics are crucial for various conditions such as cardiovascular problems, autoimmune diseases, and inflammatory disorders. There is an ever-growing need to develop diagnostic tools sensitive enough to detect and isolate minute levels of cancer markers. Researchers are increasingly exploring non-invasive alternatives to surgical biopsies that enable quick and reliable detection.

Plasmonic biosensors have been reported for analyte detection in biological matrices. Surface plasmon resonance sensor devices (SPR) have limited efficacy due to the following three reasons: (i) the lack of functionalization strategies that can fight and deter non-specific binding in complex biological systems; (ii) the lack of cost-effective fabrication methodologies for batch production with customizable detection assay; and (iii) device integration is minimal, as most reported studies focus on elemental or structural studies rather than the system.

The lack of label-free non-invasive detection tools for translational cancer research has spurred scientists to delve into real-time spectral fingerprinting at the early stages of tumor growth. Low molecular weight cancer biomarkers, such as exosomes (size: 30–150 nm), micro-RNAs (6–8 nm), and circulating tumor DNA (30–60 nm), are difficult to isolate and require high specificity to detect in blood samples. Nanophotonic sensors detect refractive index changes in the electromagnetic wave supported by the optical structure.

Hyperbolic dispersion materials are anisotropic metamaterials. These artificially designed materials’ extreme sensitivity and selectivity towards biological analytes stems from principal permittivity components with opposite signs (εzz = ε_┴_ and εxx = εyy = ε_ǁ_).

The isofrequency counter (IFC) plays an important role in controlling the interaction between light and matter. Indefinite media (HMMs), a special type of anisotropic metamaterial, forms an open hyperboloid which allows drastic control over electromagnetic waves. Hyperbolic dispersions can be tuned with artificially engineered structures allowing them to produce all-angle negative refraction, collimation, splitting, near-perfect absorption, and abnormal scattering. HMMs support surface plasmon polaritons (SPPs) and bulk plasmon polaritons (BPP) in the effective medium limit propagating inside the material but are evanescent and decay away exponentially in the superstrate. BPP modes are nonradiative with infinitely large momentum and can only be excited using a momentum coupler, such as a prism, or grating, such as surface plasmon polaritons. BPP modes of both type I (ε∥>0,ε⊥<0) and type II (ε∥<0,ε⊥>0) can be excited using either coupling technique, showing a high-quality factor (Q) resonance ideal for developing ultrasensitive multimode biosensor [2,5,6,9,12,13,14,15,16,17].

Hyperbolic metasurfaces (HMs) have gained popularity lately for their ability to detect and separate biomolecules due to topological transitions generating a strong electrical and magnetic dipole moment. Metasurfaces are extremely anisotropic two-dimensional (2D) nanopatterned structures that control the local phase and amplitude with the help of dielectric or plasmonic resonators. Thanks to extraordinary abilities, such as beam steering and wavefront engineering, dielectric metasurfaces offer a label-free biosensing platform that can sense local refractive index (RI) changes by individual biomolecules in the NIR and visible wavelength range. Metasurfaces can be combined with HMMs; metasurface grating-coupled HMM (GC-HMM) sensor performance depends on a couple of conditions. When a wavevector of light is identical to the parallel wavevector of surface plasmons, diffraction orders are evanescent fields instead of propagating waves, allowing control of light-matter interactions at the nanometer scale in biological matter [1,2,3,4,5,6].

This review highlights Hyperbolic plasmonic biosensors for cancer biomarker detection. We discuss in detail the significance of label-free point-of-care devices and compare nanofabrication design strategies, cost-effectiveness, and surface functionalization challenges in an effort to pave a pathway for better cancer screening.

## 2. Hyperbolic Metamaterials (HMMs) Design Strategy

Among the most reported Hyperbolic Metamaterial designs, the most profound metal-dielectric combinations exhibiting dispersion relationships are (i) multi-layered thin films and (ii) nanowire structures. Materials selection is crucial as it governs extraordinary optical properties such as relative magnetic permeability (µ_r_), relative electrical permittivity (ε_r_), and negative refractive index.

### 2.1. Materials Selection

Indefinite media or hyperbolic metamaterials (HMMs) are artificially engineered anisotropic materials in which the out-of-plane dielectric component εzz = ε⊥ has the opposite sign of the in-plane dielectric component εxx=εyy=ε||, resulting in unique hyperbolic dispersions. At UV frequency, Al, Au, and Ag perform better in layer formation.

Alumina does not perform well as the dielectric layer due to its light-absorbing property at visible frequency, whereas TiO_2_ performs much better; plus, its dielectric constant is relatively close to that of the metal layers. Au or Ag are excellent choices for metallic layers or nanowires because their plasmonic resonance is in the visible region. Therefore, multilayer HMMs composed of Ag/TiO_2_, Au/TiO_2_, Ag/MgF_2_, and Ag/Ti_3_O_5_ show hyperbolic dispersions, similar to Au and Ag. TiN is also a plasmonic material in the visible region and shows similar optical properties and thermal stability, making it a suitable candid choice for HMMs. Metals have been shown to perform poorly in applications that demand high-temperature stability primarily due to the electron-phonon interaction that degrades HMM performance. Heavily doped oxide semiconductors, which have a plasmon resonance in the IR spectrum, show tremendous potential.

**Figure 1:**
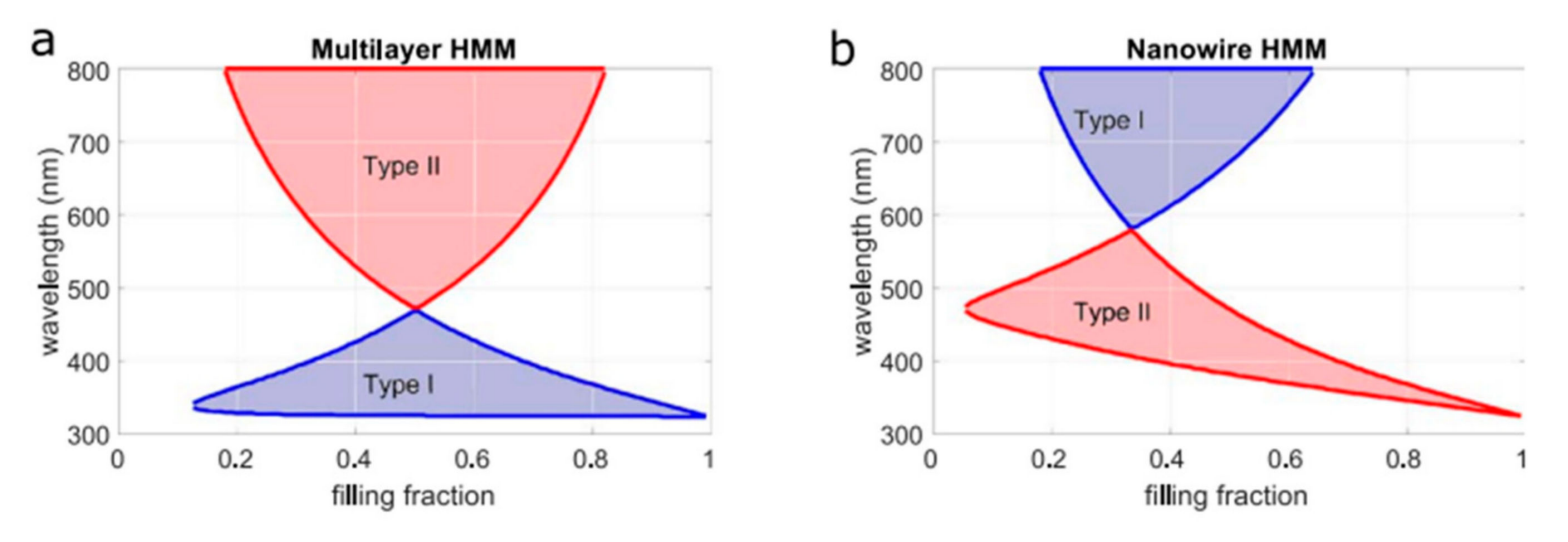
Dispersion phase diagram. (**a**) multilayered Ag/TiO_2_ (**b**) Nanowire Ag/TiO_2_. Adapted with permission from Ref. [7], 2018, Simpson, Robert Edward; Lu, Li; Kandammathe Valiyaveedu, Sreekanth.

Prominently discussed material combinations in the Mid-IR region are either multilayered, stacked, or, in some cases, HMM nanowires have been reported; examples include Al: ZnO/ZnO, InGaAs/AlInAs, and SiC as shown in Table 1. Graphene is another exciting material that demonstrates elliptical to hyperbolic dispersion for THz frequencies. Titanium is yet another favorable choice for plasmonic application when used in combinations with nitrides and oxides due to its excellent thermal stability for higher temperature phase transition material application because of its stability in crystalline or amorphous forms. Some examples of combinations include Ag/TiO_2_ and Au/TiO_2_. As shown in Figure 1 below, the multilayered or nanowire filling fraction can tremendously change the hyperbolic dispersion spectral range [7].

### 2.2. Fabrication Techniques

**Figure 2:**
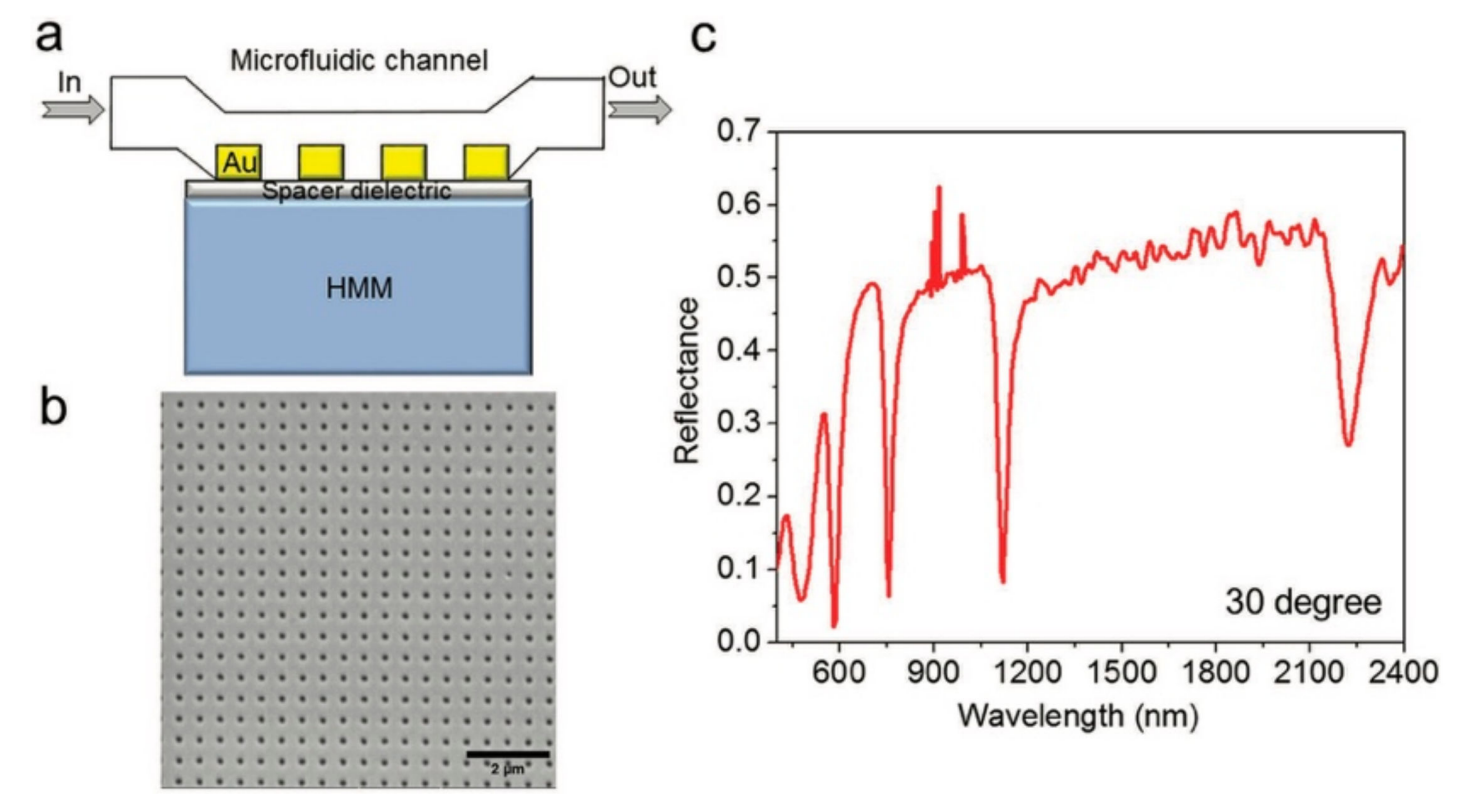
(**a**) HMM microfluidic biosensor. (**b**) Two-dimensional gold diffraction grating (period: 500 nm; hole diameter: 160 nm). (**c**) Au/Al_2_O_3_ HMM coupled grating at 30° angle of incidence spectroscopic resolution 2 nm [7,8,9,10,19,20]. Adapted with permission from Ref. [11]. 2016, Giuseppe Strangi, Nicole F. Steinmetz, Umut A, Gurkan, et al.

HMM-based ultrasensitive biosensors have evolved to a great extent; nanofabrication has enabled the development of novel metamaterials that can outperform current limitations in plasmonic sensors. In order to manufacture a high figure of merit (FOM) ultra-sensitive point-of-care device that does not rely on bulky instrumentation, we first need to overview and compare recent developments in fabrication techniques. Here, we report design strategies that have recently been studied. K.V. Sreekanth et al. developed anisotropic HMMs comprising 16 alternating thin film layers measuring 30 nm and 16 nm for Al_2_O_3_ and gold onto micro slides using electron beam evaporation at a rate of 0.3 A/s. A 2D gold diffraction grating was deposited by e-beam lithography on top of HMMs to excite High-k modes. Al_2_O_3_ (20 nm) spacer layer was built to improve coupling between HMMs and diffraction grating, demonstrated in Figure 2 [7,8,9,10,19,20].

Another way of fabricating metamaterials involves using a nanowire mesh formation instead of expensive nanofabrication methods such as e-beam lithography. Nanowires are made by electrochemical deposition. Au and Ag are grown inside a porous alumina membrane, resulting in an extremely high aspect ratio. Carbon nanotubes (CNT) have also been used in designing metamaterial platforms.

**Figure 3:**
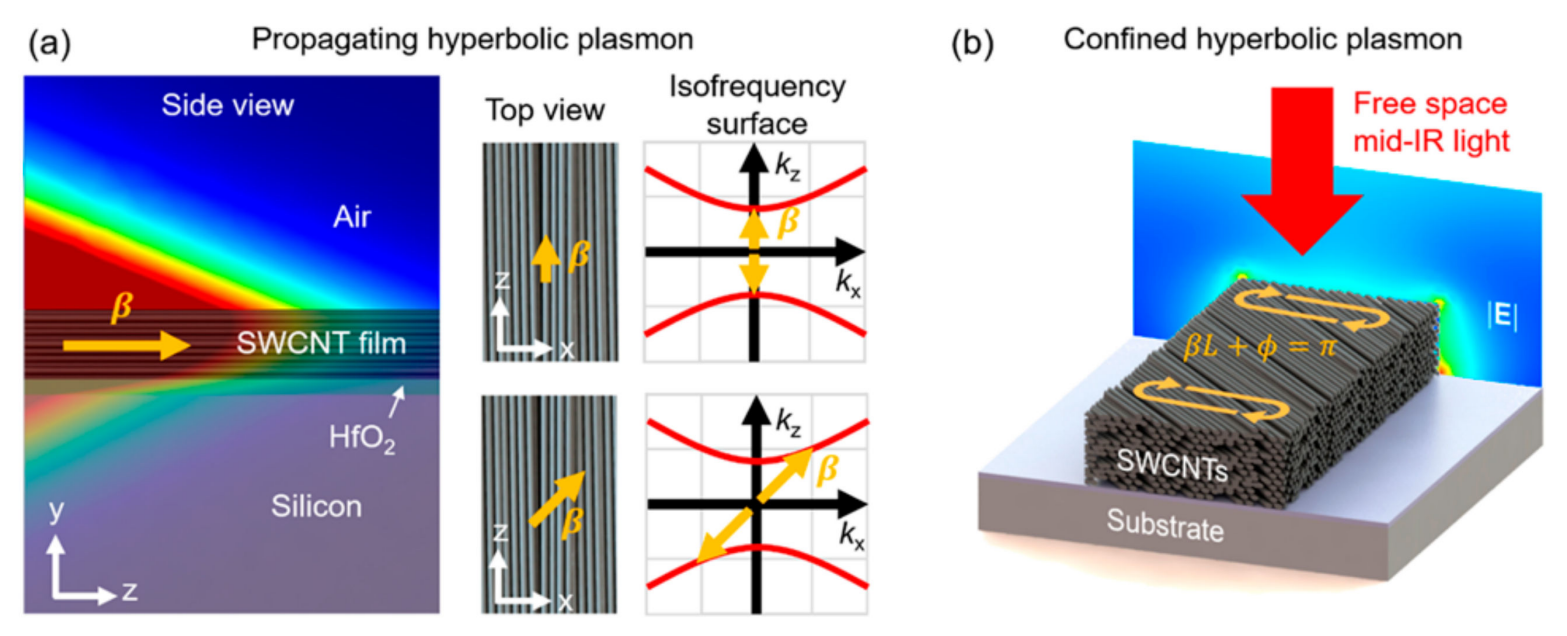
Hyperbolic plasmonic. (**a**) Side view: 95 nm thick SWCNT film on HfO_2_ and Si substrate in air. Top view: large modular wave vector in-plane at an angle to the SWCNT axis. (**b**) Nanoribbon resonator with compact hyperbolic plasmons. Adapted with permission from Ref. [21]. 2019, Roberts, J.A.; Yu, S.-J.; Ho, P.-H.; Schoeche, S.; Falk, A.L.; Fan, J.A.

A horizontally aligned single-walled carbon nanotube (SWCNT) can support in-plane hyperbolic propagations, which is quite challenging to achieve in the stacked layers but is excellent for designer HMMS shown in Figure 3. The HMM-SWCNT system was developed via a vacuum filtration technique consisting of 2/3 semiconducting and 1/3 metallic films deposited onto a Si substrate with a thin hafnia overlayer. SWCNT films show type I HMM behavior with negative permittivity in the nanotube alignment axis and positive permittivity along other axes across wide infrared ranges [21].

**Figure 4:**
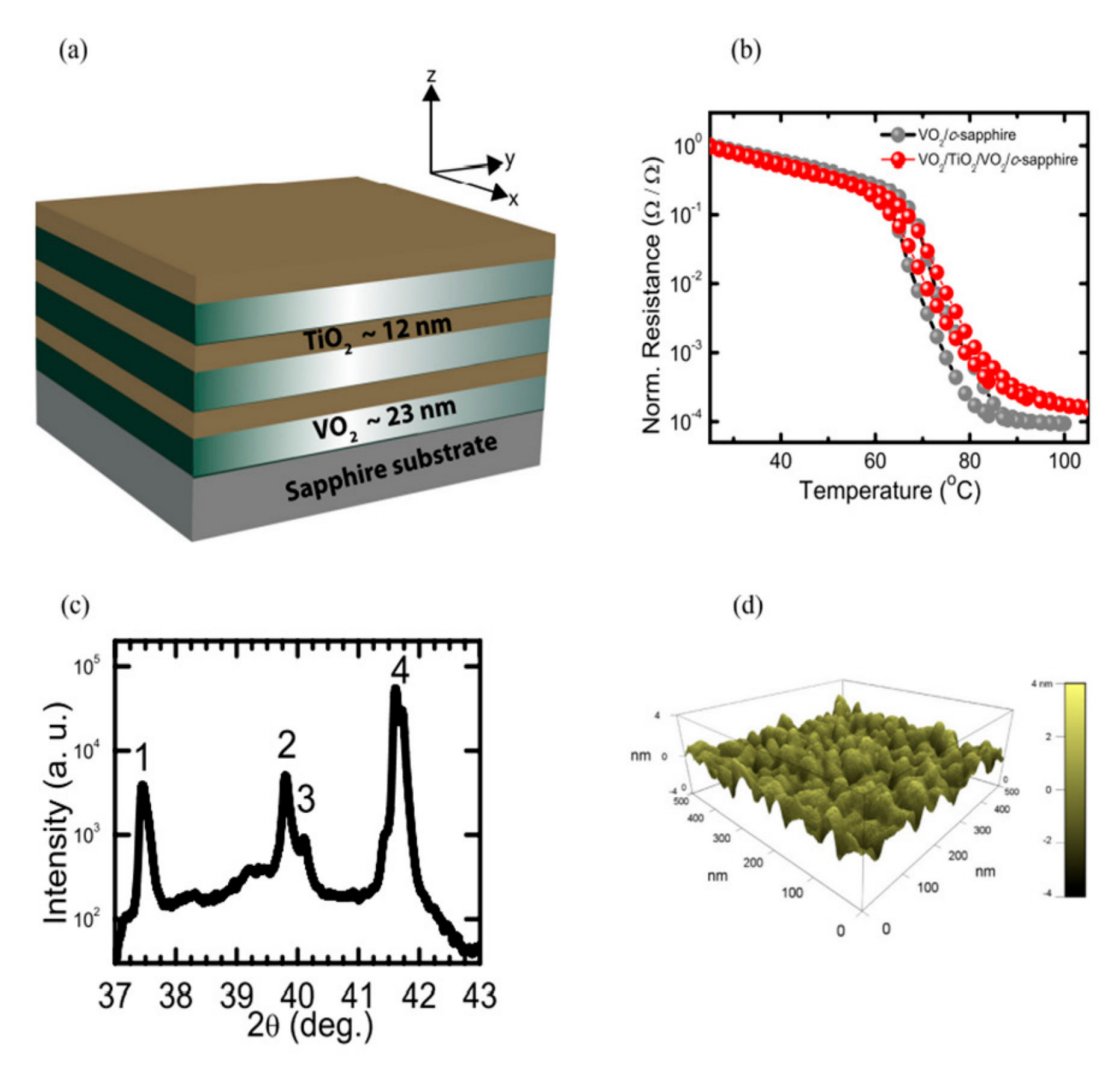
(**a**) Multilayer HMM. (**b**) Electrical resistance graph VO_2_/c-plane sapphire versus TiO_2_/VO_2_ structure on sapphire. (**c**) X-ray diffraction plot (1) TiO_2_ (004). (2) TiO_2_ (200). (3) VO_2_ (020), and (4) Al_2_O_3_ (00012). (**d**) TiO_2_/VO_2_ on sapphire with surface roughness 2.2 nm and thickness ~70 nm. Adapted with permission from Ref. [18]. 2014, Harish N.S. Krishnamoorthy, You Zhou, Shriram Ramanathan, et al.

Krishnamoorthy et al. discussed transition metal-oxides, such as vanadium dioxide. Beyond the critical temperature, its structure changes to a tetragonal rutile dropping electrical resistance ideal in tunable hyperbolic metamaterials since the mostly dielectric constant is fixed. Titanium dioxide and vanadium dioxide were carefully deposited onto sapphire using the magnetron sputtering technique in a controlled argon environment, whereas the growth rate of thin films was adjusted by variable angle spectroscopic ellipsometry (VASE) and X-ray reflectivity (XRR) readings. VO_2_ experiences insulator-metal transition at temperatures above 130 C, resulting in hyperboloid iso-frequency surface transitions for wavelength beyond 1566 nm illustrated in Figure 4 [12,18].

**Figure 5:**
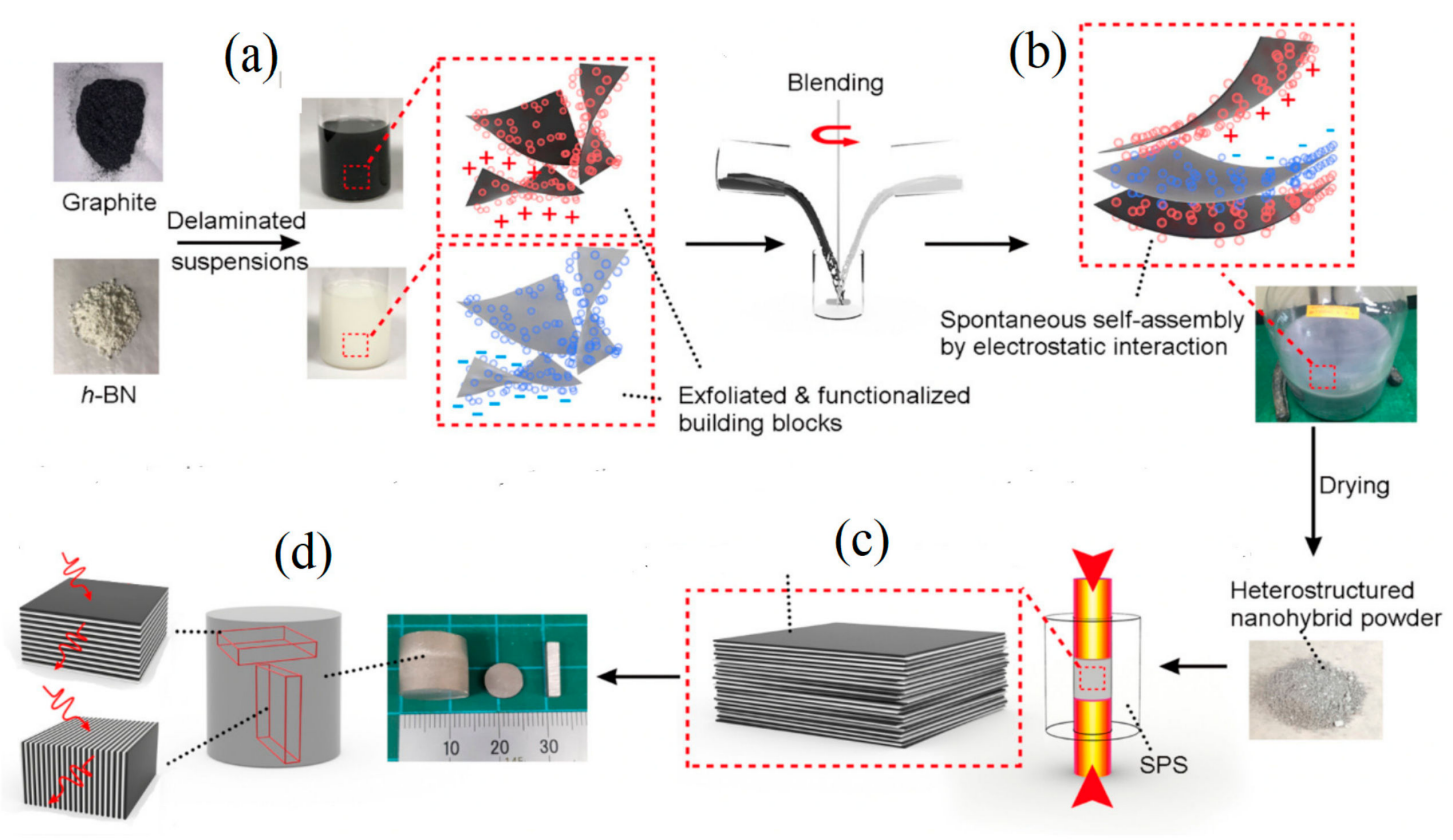
(**a**). Representation of scalable bulk heterostructure nanohybrid hyperbolic metamaterials. (**a**) Natural hyperbolic metamaterial exfoliated and functionalized. (**b**) Spontaneous self-assembly by electrostatic interaction followed by drying to form heterostructure nanohybrid powders. (**c**,**d**) Transforming powders into bulk phase with highly ordered internal structure via high temperature and pressure application. Adapted with permission from Ref. [12]. 2021, Myeongjeong Lee, Eunsil Lee, Sunae So, et al.

A simple bottom-up synthesis route was developed by Myeong Lee et al.: a natural bulk hyperbolic metamaterial with tunable properties in both in-plane and out-plane directions. Hexagonal-boron nitride (h-BN) is a natural metamaterial known for exhibiting hyperbolic properties in mid-infrared to long-infrared spectral ranges. Self-assembled heterostructures consisting of H-BN and graphite/graphene nanolayers were fabricated, displaying modulation in both types I and type II hyperbolic resonance modes, followed by disintegration/delamination of bulk HMM in distinct, well-defined thickness, namely h-BN nanoflakes (BNNF thickness ~80 nm), h-BN nanosheets (BNNS thickness ~16 nm), exfoliated graphite (EG, ~18 nm), and few-layer-graphene (FLG, ~7 nm). Afterward, the respective building blocks mixed with SDS and polyethyleneimine (PEI) to form suspension nanohybrid heterostructures were paired, driven by a self-assembly process (EG and BNNF combined, FLG, and BNNS) as shown in Figure 5 [12].

Traditionally, HMMs are made by stacking alternating layers of metal and dielectric; however, metals are prone to current losses, whereas modulating their permittivity is also challenging. In order to offset this issue, Qin wang et al. used a new kind of 3D Dirac semimetals which offers higher charge mobility than graphite and ease of modulating permittivity by changing fermi energy [2]. Dylan Lu et al. reported using a magnetron sputtering technique to fabricate multilayers of silver (Ag) and silicon (Si) onto glass at ambient conditions; 10 nm thick layers of Ag and Si in a pair of 15 were produced. A 5 nm capping layer of Si was applied to prohibit Ag oxidation and quenching [22].

**Figure 6:**
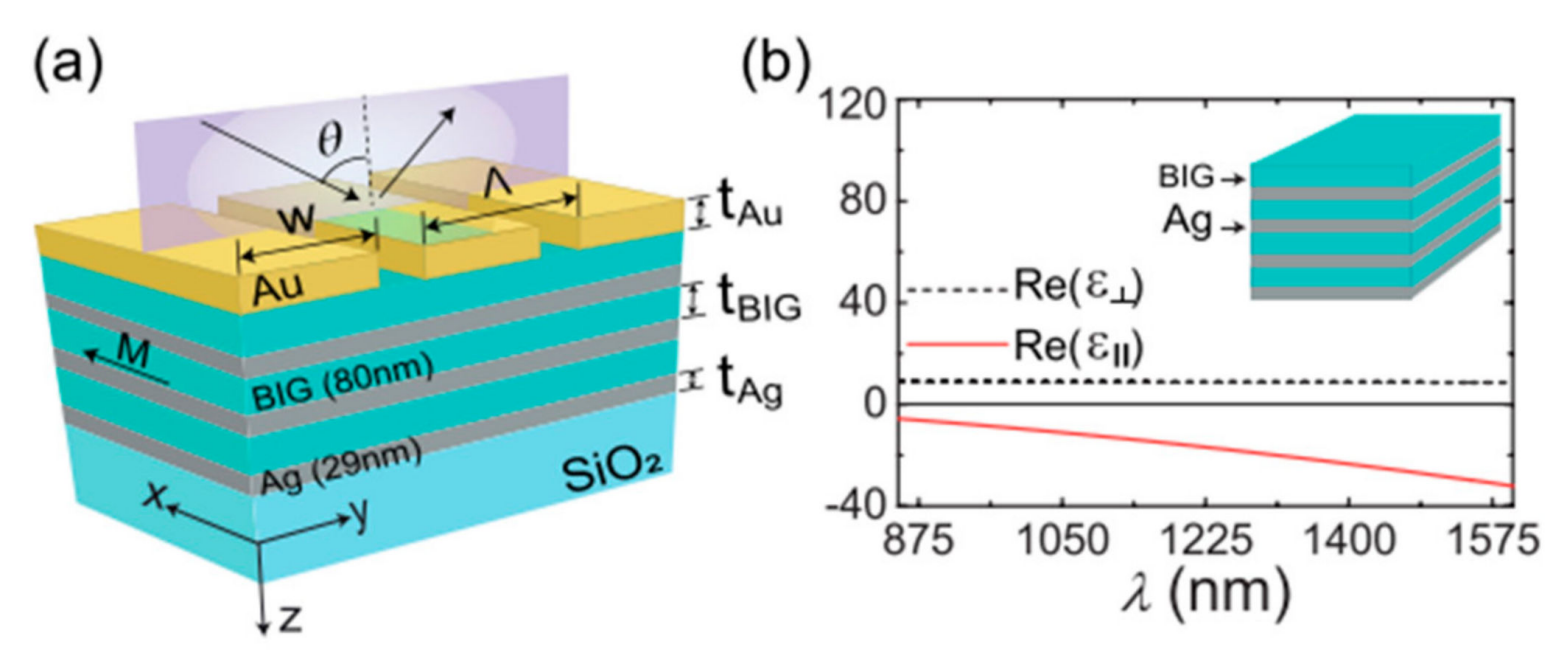
(**a**) Magnetooptical hyperbolic metamaterial (MO-HMMs) made of alternating slabs Ag/dielectric Bismuth-iron garnet onto silicon dioxide substrate. Gold grating to instigate BPP modes. chematic of the proposed architecture, comprising altering metal/dielectric (Ag/BIG) layers on SiO_2_ substrate. A periodic one-dimensional Au grating is used to couple the incident light to the BPP modes in the multilayer structure. (**b**) Effective permittivity ε∥ and ε_┴_. Adapted with permission from Ref. [23]. 2022, Brayan F. Diaz-Valencia, Nelson Porras-Montenegro, Osvaldo N. Oliveira Jr., et al.

Brayan F. Diaz-Valencia et al. developed magneto-optical hyperbolic metamaterials (MO-HMMs) demonstrated in Figure 6; based on ultranarrow fano-like TMKO curves obtained through MO modulations of BPP modes in nanostructured HMMs. The plasmonic biosensing platform offers label-free detection of low molecular weight biomolecules on the order of picomolar [23].

**Figure 7:**
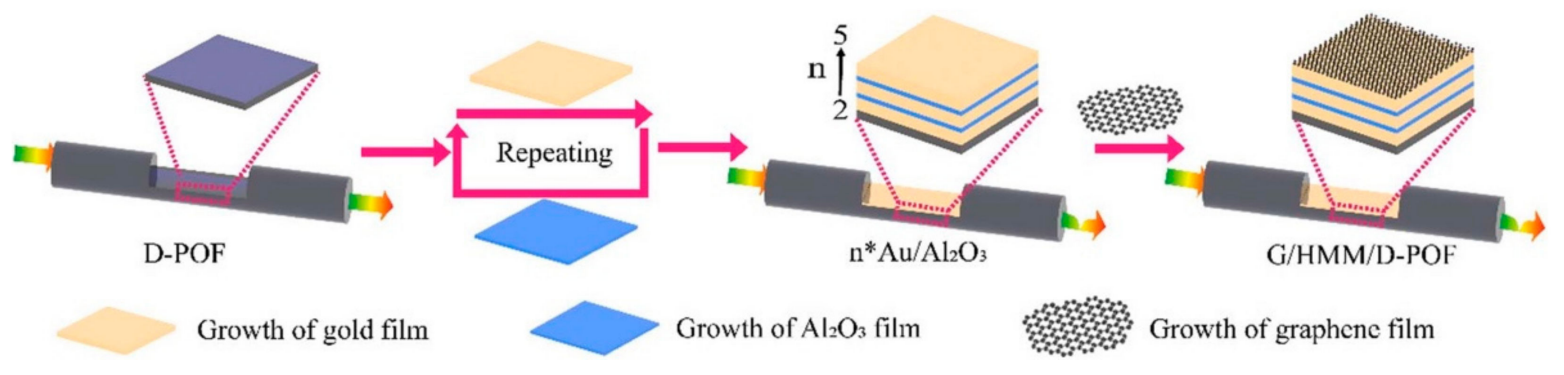
Preparation steps for G/HMM/D-POF. Adapted with permission from Ref. [24]. 2021, Li, C.; Gao, J.; Shafi, M.; Liu, R.; Zha, Z.; Feng, D.; Liu, M.; Du, X.; Yue, W.; Jiang, S.

Can Li et al. report a high-resolution detection limit from 10 pM to 100 pM with a sensitivity value up to 4461 nm/RIU through an optical fiber-based surface plasmon resonance (SPR) for measuring DNA hybridization kinetics. Optical fiber sensors offer a miniaturized biosensing platform compared to traditional SPR sensors with a built-in Kretschmann configuration. The layout of a 3D HMM optical fiber composite sensor shown in Figure 7 represents an overlapping scheme: Au/Al_2_O_3_ (HMM), graphene film, and a D-shaped optical fiber (D-POF) [(D-POF) (G/HMM/D-POF)]. Tunable HMM fabrication design involves the step-by-step layering of gold and aluminum oxide such that Au is divided into n layers varying from 2–5, respectively. The gold layer thickness was reportedly 50 nm; in contrast, the entire functional layer was compressed under 80 nm. Thermal evaporation was used to deposit gold onto POF at a rate of 0.7 A°. s^−1^ (1 A = 0.1 nm) simultaneously. The plasmonic efficacy of graphene was improved by the gold layer on HMM. Oxidized alumina film thickness was kept at 6 nm (*Au/Al_2_O_3_).

Later, the HMM/D-POF surface was immersed for an hour in a PAH solution (1 g·L^−1^), followed by 5 h reaction in a graphene oxide dispersion (0.2 g·L^−1^; flake diameters 50–150 nm), and 1 h in hydrazine (N_2_H_4_ 50%) to obtain the G/HMM/D-POF structure, followed by washing with DI and drying with nitrogen. G/HMM/D-POF was modified for DNA detection with 1 micromolar PBAS solution for 4 h, followed by washing with DMSO and de-ionized water; finally, probe aptamer was fixed with -NH_2_ by inserting the sensor into 1 micromolar solution for 4 h and then rinsing with DI water and PBS (illustrated in Figure 7) [24].

## 3. Performance Evaluation

The label-free point-of-care device requires an extensive evaluation to articulate the relevant information, which is crucial in developing and exploring novel design approaches. K.V. Sreekanth et al.’s work represents a novel device capable of supporting highly confined bulk plasmon modes (BPP) in HMM. High spectroscopic and angular resolutions of 0.03 nm and 0.01° were observed; above a 500 nm wavelength, Q-factor modes of 29.5, 26, and 23 at 1120 nm, 755 nm, and 580 nm were calculated.

**Figure 8:**
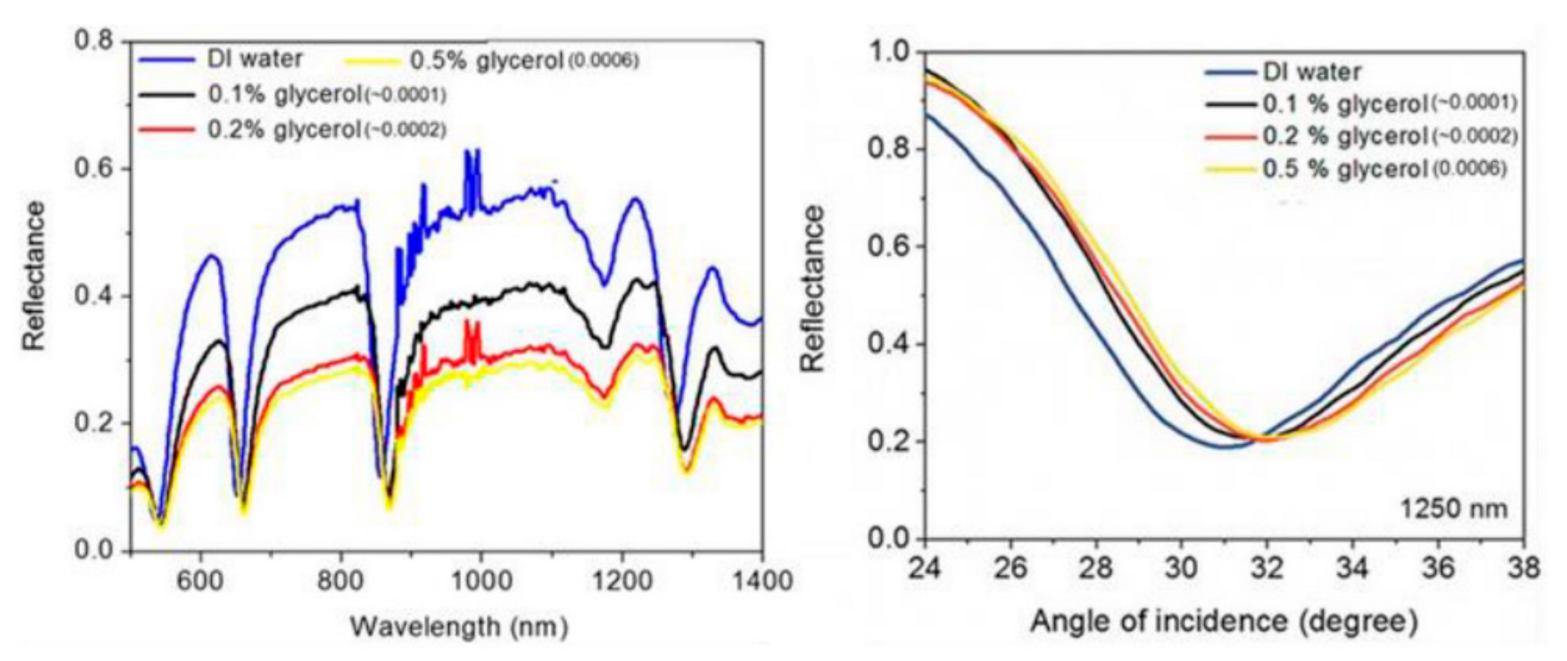
(**Left**): Reflectance spectra at 30°angle, glycerol in distilled water (0.1–0.5% *w*/*v*). (**Right**): Angular interrogation scheme, reflectance spectra at 1250 nm [4,7,8,9,10,19,20,25]. Adapted with permission from Ref. [9]. 2017, Sreekanth, K.V.; ElKabbash, M.; Alapan, Y.; Ilker, E.I.; Hinczewski, M.; Gurkan, U.A.; Strangi, G.

A standardized calibration test was conducted by introducing multiple concentrations of glycerol in a microflow channel (volume 14 × 2 × 0.05 mm^3^), which gave valuable insight; as the weight concentration increases, a red shift in resonance wavelength is observed along with a decline in the quality factor, indicating a decrease in the performance of the biosensor device. However, even with a low concentration of glycerol, the biosensor device picked up minute refractive index shifts. As shown in Figure 8, a considerable shift was observed at 0.1% (*w*/*v*) glycerol. To identify the detection range difference in the refractive index between DI water and 0.5% (*w*/*v*), glycerol was considered, which was found to be at around 0.0006. Maximum spectral and angular sensitivity recorded at 1300 nm were around 30,000 nm/RIU and 2500/RIU, whereas lower end values for spectral and angular sensitivity recorded at 530 nm were 13,333 nm/RIU and 2333/RIU [4,7,8,9,10,19,20,25].

The sensor device shows different sensitivities for each mode. There is flexibility in the selection of a particular mode for the identification of specific biomolecules. In other words, using a lower sensitivity mode detects higher molecular weight biomolecules and a higher sensitivity mode detects lower molecular weight biomolecules [4,10,19,20,25].

### 3.1. Comparison of HMM-Based Biosensors

Tunable metamaterials are next-generation biosensors that outperform traditional plasmonic sensors because of their extraordinary sensitivity and the ability to detect low-concentration analytes in clinical samples. Despite all the advantages, label-free detection and quantification are still challenging due to the lack of cancer biomarker studies performed on HMMs. Herein, we will try to shed light on the most recent HMM-based point-of-care (POC) systems. Sreekanth et al. have performed pioneer studies on extremely sensitive hyperbolic dispersion meta-surfaces. In 2019, a dynamic biosensor was developed for low and high molecular weight analyte detection with tunable properties fabricated by combining titanium nitride (TiN) and stibnite (Sb2S3) operating in a visible frequency range suitable for label-free selective detection as real-time demonstration shows binding of biotin at 1 × 10^−12^ M concentration. The refractive index (RI) sensitivity was magnified via Gosse-Hanchen (G-H) shift interrogation scheme to a much greater extent (10 fM to 1 µM), allowing a reported detection limit of less than 1 pM.

**Figure 9:**
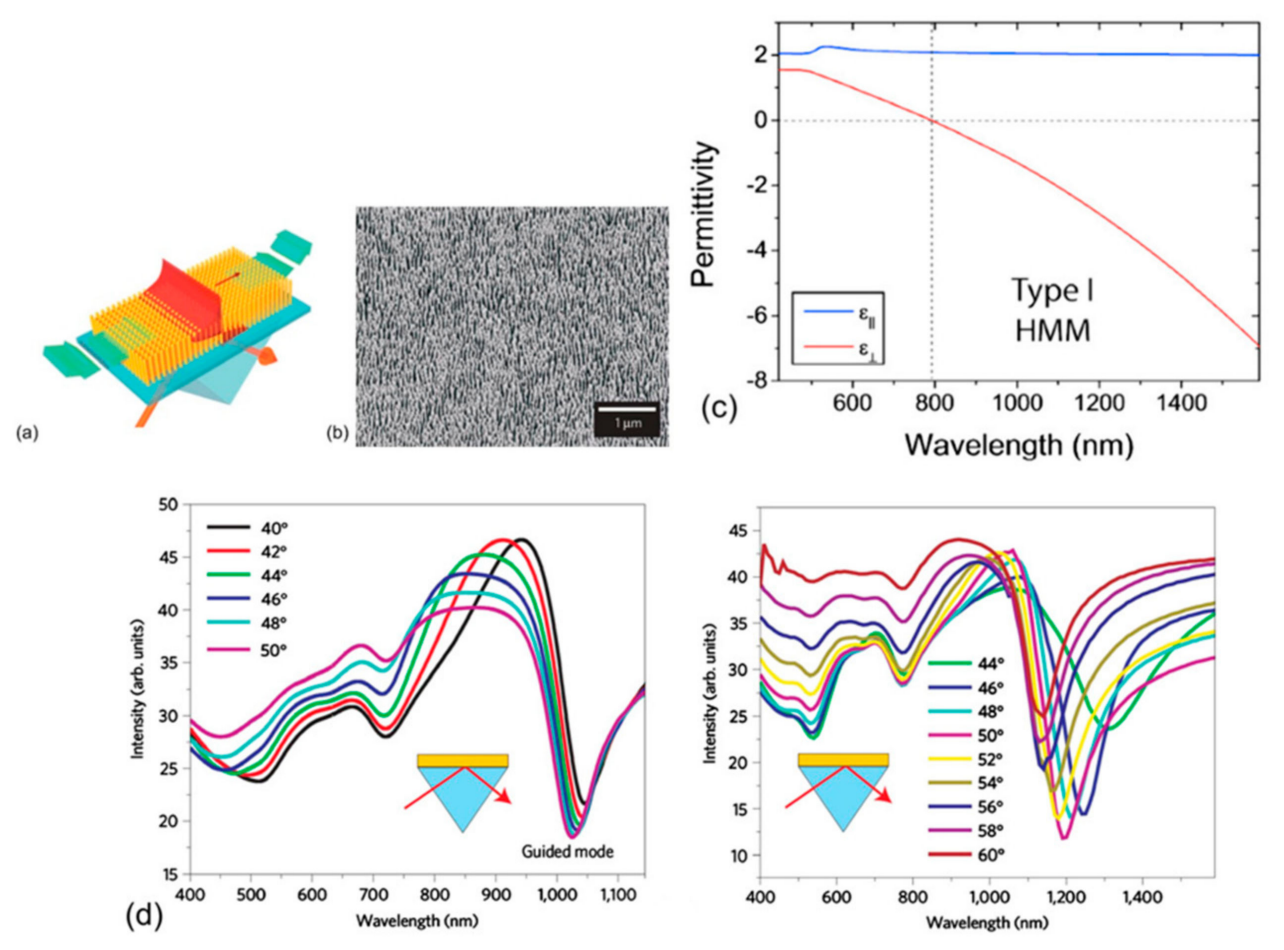
(**a**) Gold nanopillar representation. (**b**) SEM image of nanorod array. (**c**) Nanorod array effective permittivity tensor in water. (**d**) Attenuated total internal reflection in air (n = 1) and water (n = 1.333). Reprinted with permission from Ref. [4]. 2019, Pengcheng, Huo; Si, Zhang; Yuzhang, Liang; Ting, Xu.

Both types I and II HMMs are frequently reported for biosensing applications. Kabashin et al. improvised LSPR sensors with type I HMM gold nanopillars based on traditional SPP biosensor technology but exhibited enhanced sensitivity to minute refractive index variations in target analyte. For example, a change of 10–4 RIU causes a resonance shift by 3.2 nm without structural optimization (Figure 9). In comparison to LSPR sensors, the minimum sensitivity of 32,000 nm/RIU exceeds two orders of magnitude, whereas a FOM (figure of merit) value of 330 has been reported [4,7,8,9,10,19,20,25].

Earlier in 2016, Sreekanth et al. reported a type II HMM capable of detecting 244 Da molecular weight biotin, which is an excellent model for cancer biomarker studies. The multi-layered metamaterial consisted of a two-dimensional (2D) subwavelength Au diffraction grating multimode biosensor with a period of 500 nm and hole diameter of 160 nm, demonstrating six orders of magnitude sensitivity.

Regarding performance, bulk refractive index (RI) sensitivity for the most extended wavelength BPP mode in the spectral and angular scan is around 30,000 nm/RIU and 2500°/RIU. At the same time, the minimum sensitivity recorded at the shortest wavelength BPP mode is 13,333 nm/RIU and 2333°/RIU for spectral and angular scans. Microfluidic channels were incorporated to effectively relate reflectance spectra corresponding to variation in biotin concentration (10 pM–10 µM) in PBS with a 40 min break between concentrations. A 0.2 nm discreetness in wavelength sensitivity was observed as part of the real-time molecular binding event causing step formation in resonance wavelength.

**Figure 10:**
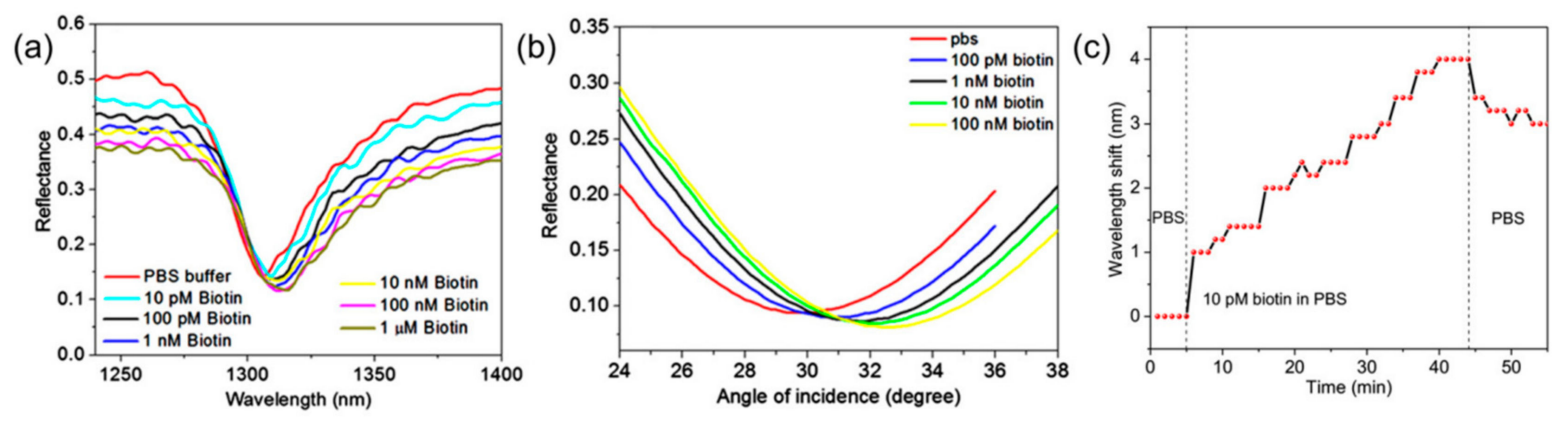
(**a**) Reflectance spectra as a function of wavelength with different percentage of biotin in PBS at 30° angles of incidence. (**b**) Reflectance spectra as a function of incident angle with different concentration of biotin in PBS at wavelength 1250 nm. (**c**) Illustration of real time binding of biotin by injecting 10 pM biotin in PBS (time interval between the two dotted line). Adapted with permission from Ref. [11]. 2016, Kandammathe Valiyaveedu Sreekanth et al.

Biotin-Streptavidin interactions directly influence spectral and angular shifts, which is clearly demonstrated in Figure 10 (refractive index value change with trends in concentrations). The above iteration gives guidelines for developing label-free point-of-care devices that can be implemented for cancer biomarker detection [2,9].

### 3.2. Metasurfaces for Molecular Biorecognition

Yesilkoy et al. fabricated a novel dielectric metasurface-based platform that supports bound states in the continuum (BIC) in the form of a super cavity mode within NIR and the visible range. The photonic device is capable of hyperspectral imaging (HIS) that can digitally sense less than three molecules per µm^2^ without enabling spectrometers.

The most significant feature of the photonic device was the adherence to a data science technique, a pixel-based thresholding method generating millions of spectra, each linked to complementary metal oxide semiconductor (CMOS) sensor pixel showing improved detection for label-free multiplexed biomarker detection. Another important design feature included a multi-resonance sensor (MRS) and a hyperspectral decoder capable of spatially indexing each spectral data from a CMOS pixel resulting in high-resolution imaging at a fixed wavelength.

**Figure 11:**
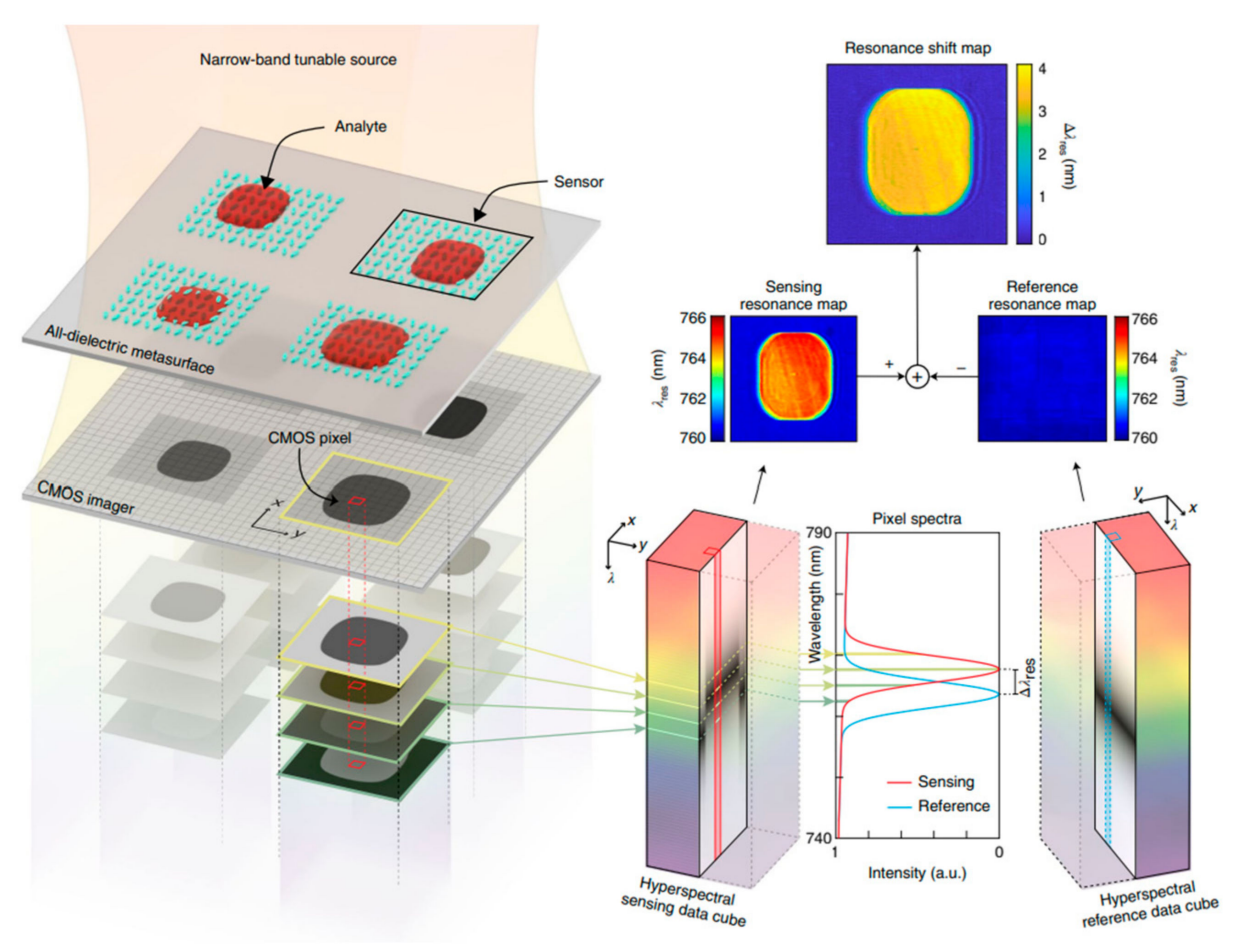
Schematic representation of hyperspectral image-based biomolecule detection using all-dielectric metasurface. Adapted with permission from Ref. [26]. 2019, Filiz Yesilkoy et al.

**Figure 12:**
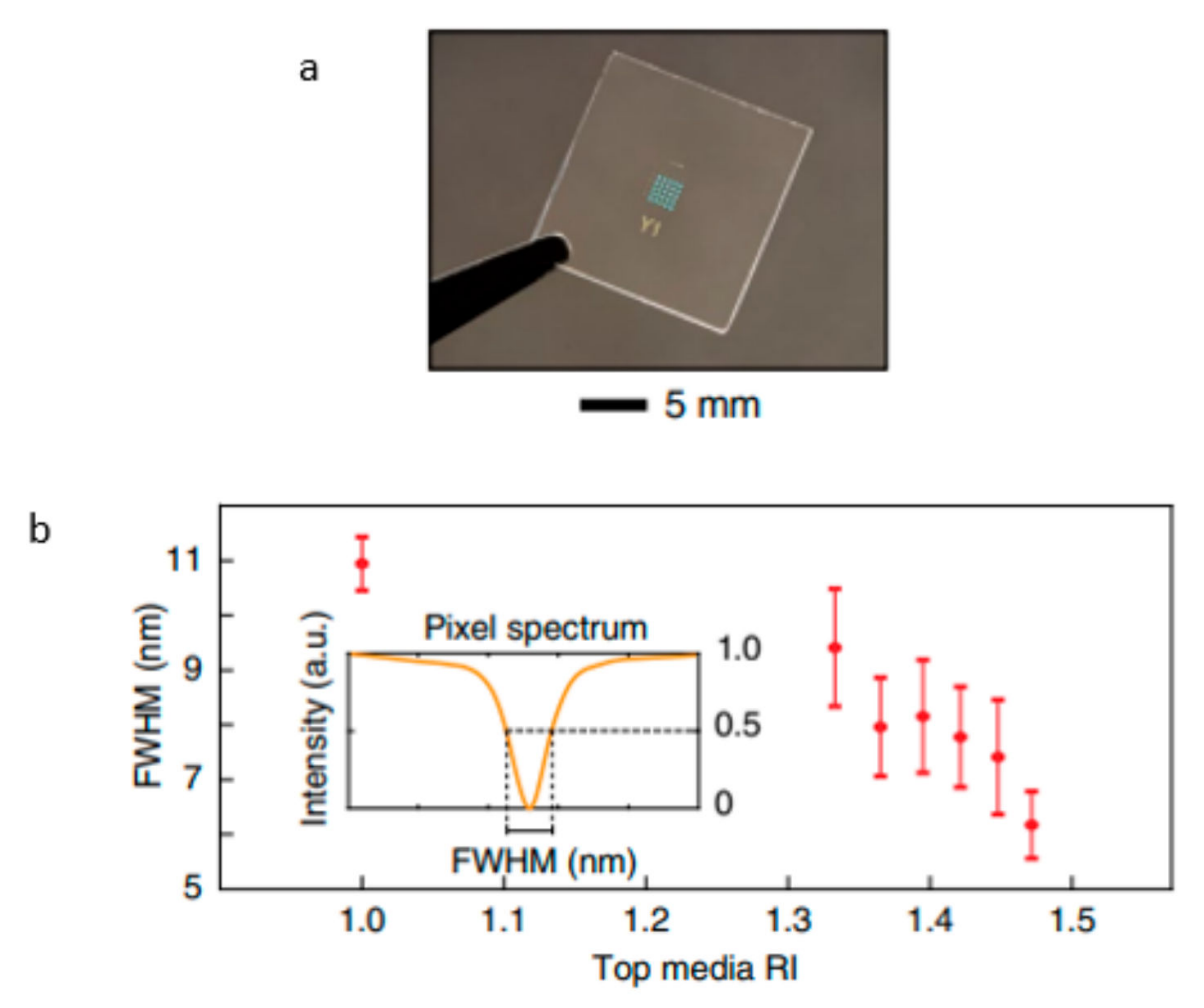
(**a**) Pictorial representation of a 5 × 5 dielectric sensor chip. (**b**) Spectral linewidth of the resonance dip measured in presence of media with different refractive indices (nm = 1–1.47), indicating Q-factors as high as 144. The error bar represents the standard deviation of FWHM measurement of 2.5 × 105 pixel spectra acquired from four different sensors. Adapted with permission from Ref. [26]. 2019, Filiz Yesilkoy et al.

As shown (Figure 11), 2 nm narrow bandwidth excitation with a high spectral resolution Δλ = 0.1 nm was used to probe metasurface response over large areas and record images using a CMOS camera. The following optical configuration paved the way for a hyperspectral data cube field of view generation consisting of spectral information to create a resonance map from the sensor array. Thousands of CMOS pixels were mapped with individual sensors (Figure 12), where a specific area on the metasurface and its corresponding resonance value are represented by an image pixel. Amorphous Silicon was fabricated via a CMOS-compatible design in tilted elliptical nano-Si pairs of bars in mirror symmetry on a 5 × 5 sensor chip. Different concentrations of glycerol (n_m_ = 1:1.47) were tested to calculate resonance dip using full-width at half maximum (FWHM) in order to find a high Q-factor (~145). BIC-type dielectric metasurfaces were perfected to endure 850 nm resonance attaining high near field enhancement 40 RIU^−1^ and a refractometric sensitivity of 263 nm RIU^−1^ [26].

## 4. Cost-Effectiveness

**Figure 13:**
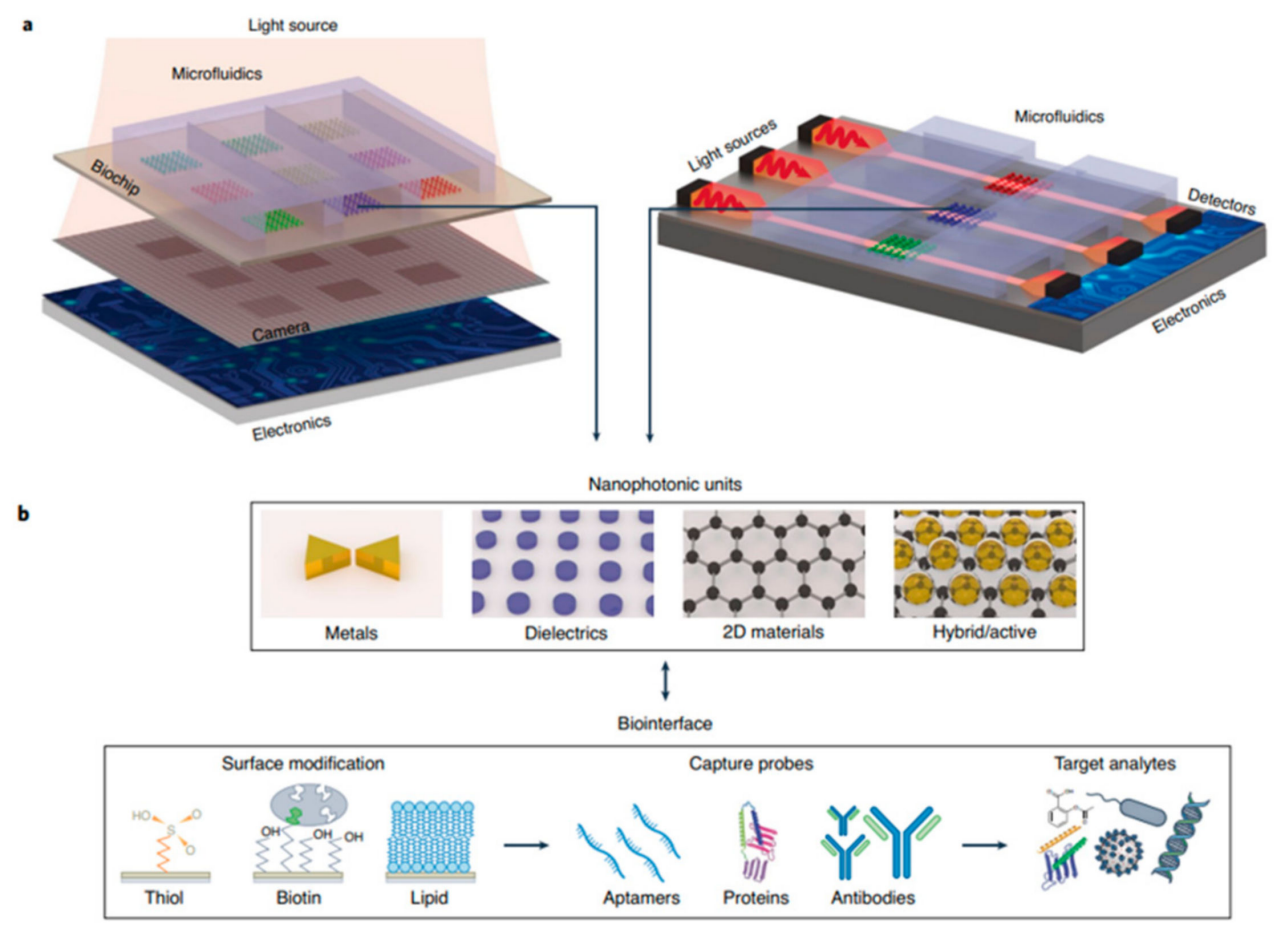
(**a**) Nanophotonic biosensor device. (**b**) Combination of nanophotonic units and surface functionalization approaches. Reprinted with permission from Ref. [27]. 2022, Hatice Altug et.al.

Point-of-care sensor devices offer compact single-use cartridge reader solutions for analyzing biological samples, avoiding specific nuances such as cross-contamination and lengthy cleaning procedures. Figure 13 illustrates a cost-effective solution using disposable cartridges that can reduce or omit the regeneration of surface functionalization, drastically improving sensor performance capability over time. The downside is the reagent cost to produce inexpensive single-use cartridges. As shown below, a nanophotonic chip can be housed in a cartridge separated from the light source and detector; later, disposable cartridges can readily be customized for detecting different analytes.

Another approach is merging photonic devices with smartphones to utilize their light sources, camera, image processing, and communication capabilities. These portable handheld sensor devices offer a flexible solution to measure a signal from patient samples, analyze data with personalized phone-based software applications and then seamlessly send wireless results to doctors. Without a doubt, the small size and portability are key features of planar biosensors, but the manufacturing cost due to complex fabrication are limiting factors. Meanwhile, nanophotonic biochips require repeated patterning of nanostructures, and electron-beam or focused-ion beam lithography are common nanostructure patterning methods; inherently costly and have low throughput limiting their commercial desirability.

There is a growing demand for alternative fabrication methods, and nanoimprint lithography is a budget-friendly alternative that requires mold to create patterns on substrate surfaces, and co-polymer lithography leverages self-assembling block co-polymers to create patterns [28].

### Surface Functionalization

Surface immobilization is a significant hurdle in biosensor development, especially when complex clinical samples, such as serum, blood, and saliva, are being tested for analyte detection. Most of these issues arise due to non-specific bindings, protein fouling, and, in some cases, unreacted easter groups found in surface-assembled monolayers. The issue becomes more critical when low-concentration biomolecules are identified. Conventional surface modification techniques include protein blockers (Bovine serum albumin or casein), detergents (Tween-20), or polyethylene glycol.

However, these methods tend to have limited reliability; novel approaches are currently being studied to understand better the inter-molecular interactions in the monolayers involved in surface-immobilized biomolecular systems. Zwitterionic polymers are an excellent example of an alternative solution to non-fouling issues, but they cannot be applied universally. Coating thickness is another constraint.

A minimum 10–20 nm thickness is required for proper analyte–receptor binding. Streptavidin-biotin or covalent binding is used for immobilization, but reversible interaction can lead to continuous biosensing. Nanophotonic biochips with multiple materials and uneven sensitivity require new functionalization approaches, such as material or site-specific methods [27].

## 5. Present Cancer Detection Methods

Infectious disease prevention and detection are one of the most dire issues in the 21st century. Each year, millions of people are diagnosed with hepatitis B virus (HBV), and many die every year due to complications related to hepatitis B. An upward cost estimated to exceed EUR 800 million per year is spent on diagnosis and treatment. Plasmonic biosensing has evolved over the years and demonstrated substantial improvements. Still, there is a long road ahead in transforming scientific breakthroughs into real-life applications. Here, we will be focusing on current biosensors used in cancer and other diagnostics.

**Figure 14:**
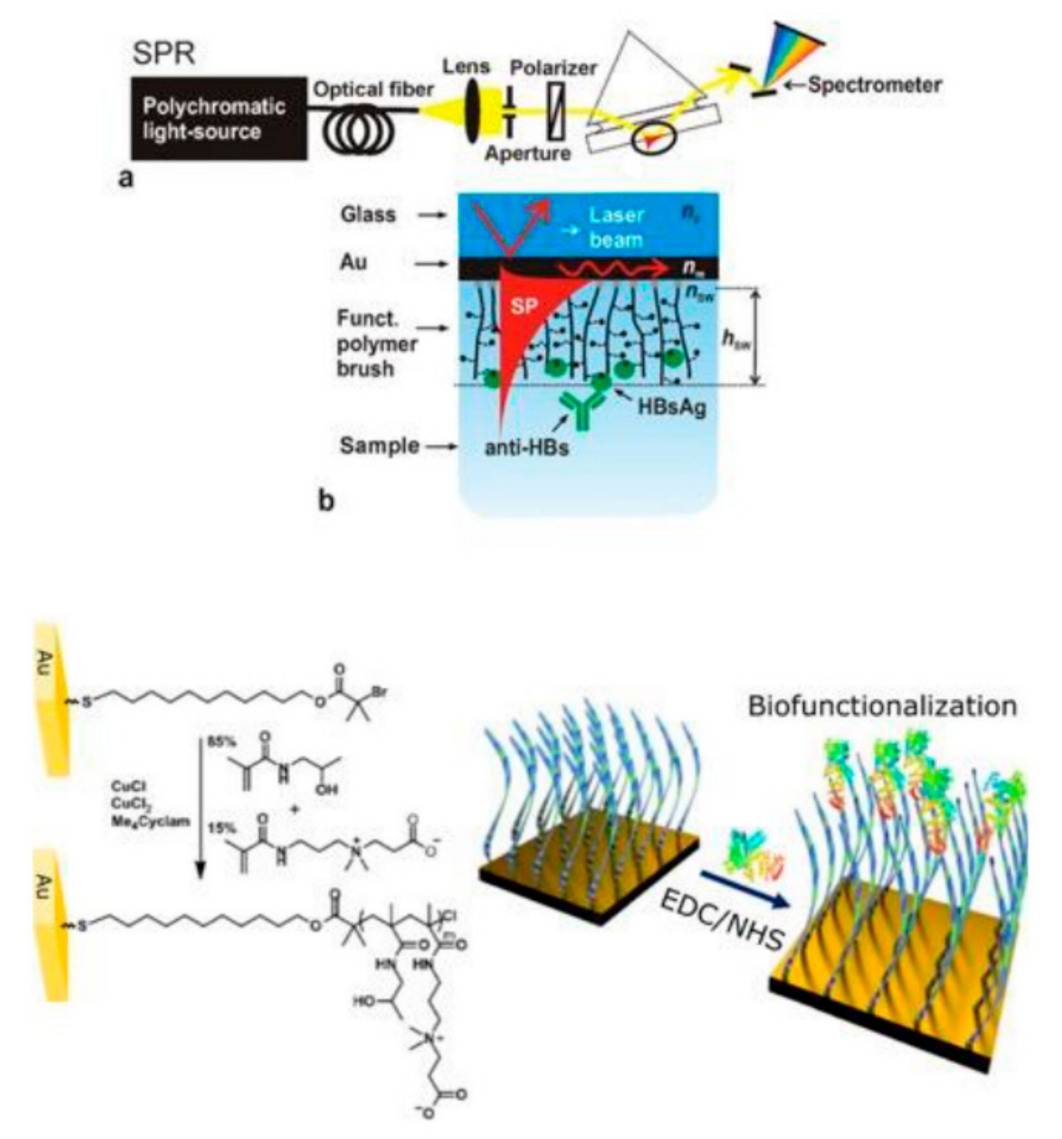
(**Top**): Surface plasmon resonance sensor chip setup (Schematic diagram of the surface plasmon resonance setup (**a**). Sensor chip with poly(HPMA-*co*-CBMAA) brush functioning as a binding matrix for direct detection of anti-HBs target analyte (**b**)). (**Bottom**): Synthesis and chemical structure of poly (HPMA-CO-CBMAA) polymer brush (**right**) and schematics of gold surface with tethered brush that is post-modified with protein ligand (**left**) Adapted with permission from reference [28]. 2016, Tomas Riedel, Frantisek Surman, Simone Hageneder, Ognen Pop-Georgievski, Christa Noehammer, Manuela Hofner, Eduard Brynda, Cesar Roriguez-Emmenegger, Jakub Dostalek.

**Figure 15:**
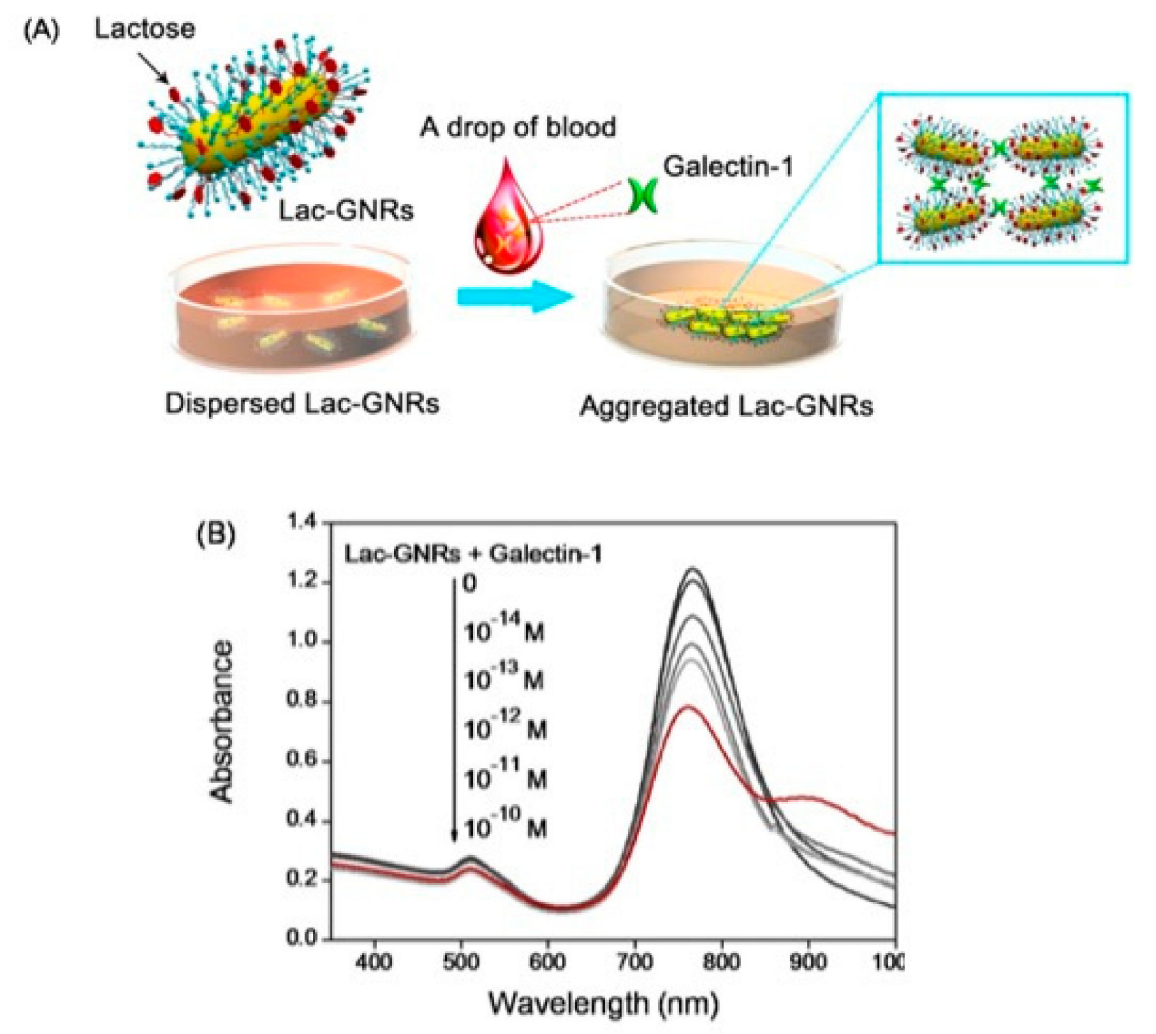
(**A**) Schematic illustration of the synthesis of Lac-GNRs; galectin-1 induced aggregation of Lac-GNRs. (**B**) Absorption spectra of Lac-GNRs. Adapted with permission from Ref. [29]. 2016, Yuetao Zhao, Liping Tong, Yong Li, et al.

Thomas Rediel et al. developed a label-free plasmonic biosensor capable of detecting the Hepatitis-B virus, one of the major causes of liver cancer, in less than 10 min (Figure 14). Different concentration ranges were tested; high (>1 IU/mL) and low range (<0.002 IU/mL) compared to ELISA. Protein fouling and other non-specific binding were prevented by poly (HPMA-co-CBMAA) brushes functionalized onto biosensors via surface-initiated radical polymerization, which performed well in blood plasma samples. Direct detection was carried out in serum for each assay. The SPR wavelength shifts or change due to anti-HBs binding were reported after a 10 min sample flow between each reading (Figure 15).

Thomas Rediel et al. developed a label-free plasmonic biosensor capable of detecting the Hepatitis-B virus, one of the major causes of liver cancer, in less than 10 min (Figure 14). Different concentration ranges were tested; high (>1 IU/mL) and low range (<0.002 IU/mL) compared to ELISA. Protein fouling and other non-specific binding were prevented by poly (HPMA-co-CBMAA) brushes functionalized onto biosensors via surface-initiated radical polymerization, which performed well in blood plasma samples. Direct detection was carried out in serum for each assay. The SPR wavelength shifts or change due to anti-HBs binding were reported after a 10 min sample flow between each reading (Figure 14).

**Figure 16:**
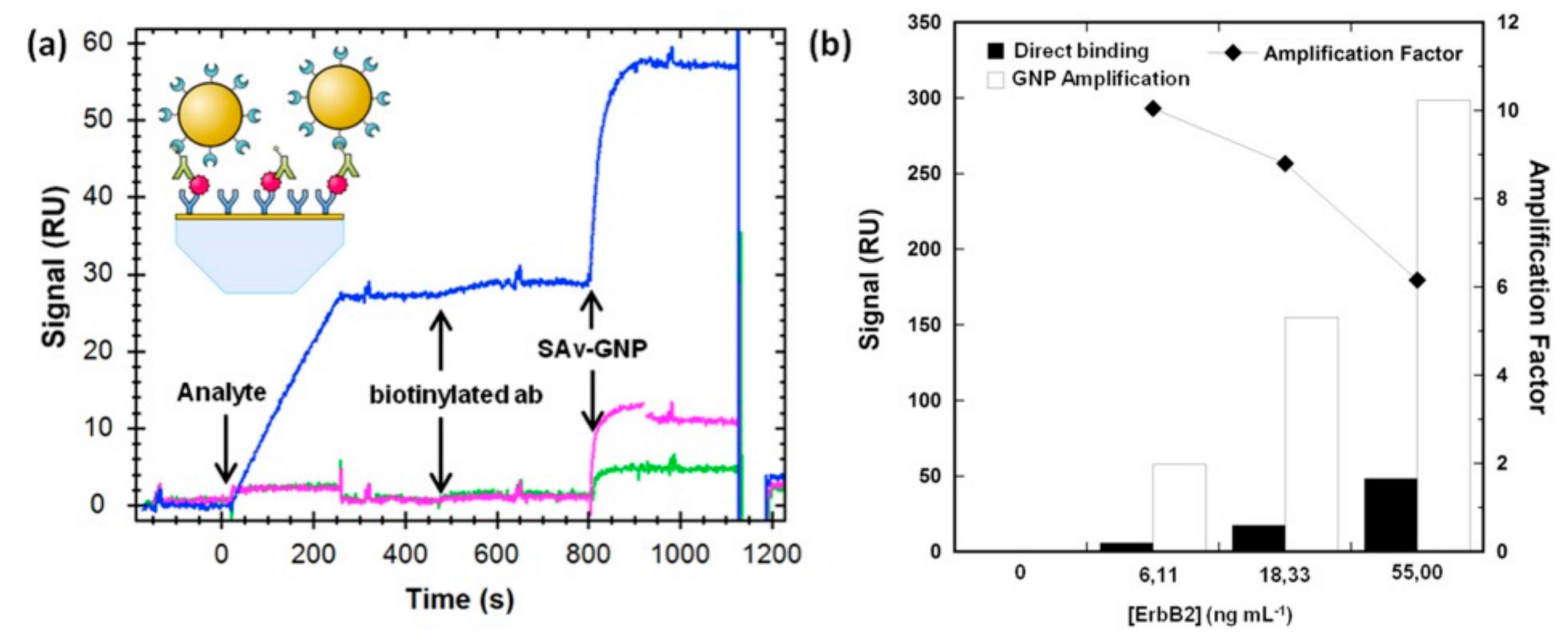
(**a**) ErbB2 solutions (0, 0.68 and 6.11 ng mL^−1^) sensogram evaluation. Top image: GNPs-amplified sandwich immunoassay. (**b**) Performance evaluation. Adapted with permission from Ref. [30]. 2016, Eletxigerra, U.; Martinez-Perdiguero, J.; Barderas, R.; Pingarrón, J.M.; Campuzano, S.; Merino, S.

Zhao et al. developed an efficient cancer detection technique based on lactose-functionalized gold nanorods (Lac-GNRs) in order to replace enzyme-linked immune sorbent assay (ELISA) Figure 15. Galectin-1, a member of the lectin family, is one of the most prominent biomarkers in tumor development and is recognizable by its unique carbohydrate-recognizing domain (CRD). Biosensor was fabricated using the longitudinal SPR technique; gold nanorods were functionalized via sulfhydryl lactose ligand (Lac-SH) adhering by Au-S reaction. The Lac-GNRs platform was utilized to perform a label-free assay; a small amount of galectin-1 was added at room temperature in a mixture of Lac-GNRs experimental protocol for an hour, as shown in the Figure 16b by a decrease in LSPR peak intensity at 770 nm. Lac-GNRs LSPR-biosensors showed high sensitivity and selectivity towards trace amount of galectin-1 biomarker detection in the range of 10–13 M [29].

Breast cancer is one of the most prevalent diseases found in women. Eletxigerra et al. developed a susceptible plasmonic sensor for detecting ErbB2 biomarkers commonly found in SK-BR-3, MCF-7, and MDA-MB-436 cancer cell lines. Gold Nanoparticle (GNPs)-based biosensor housed in a microfluidic chamber with the ability to detect the slightest changes in real-time was custom designed to measure 180 pg/mL target analyte (Limit of detection LOD) in a 50% human serum. The sensor chip surface was modified for unwanted non-specific binding by 4M NaCl and 0.1%Tween 20 in PBS, which eventually failed mainly due to the unreacted N-hydroxy-succinimide (NHS)-ester groups found on the self-assembled monolayers (SAM). Two different approaches were used for the SPR immunosensor: (i) direct-label-free binding and (ii) the sandwich format, including GNP amplification. However, label-free detection did not perform well in serum. In order to enhance sensitivity, the sandwich assay format (secondary anti-ErbB2 (biotinylated), antibody, and (Sav-GNP) streptavidin decorated gold nanoparticle) was analyzed, although the label-free characteristic was lost.

The experimental findings for ErbB2 showed LOD values 3 and 60 times lower using direct and SAv-GNP-based sandwich strategies (3.8 ng/mL and 190 pg/mL), compared to earlier ErbB2 detection via SPR-biosensor using recombinant protein-G with a detection value of 11 ng/mL (Figure 16). Due to inherent problems associated with labelled molecules, such as false positive responses, SPR-based plasmonic biosensors are becoming increasingly popular.

**Figure 17:**
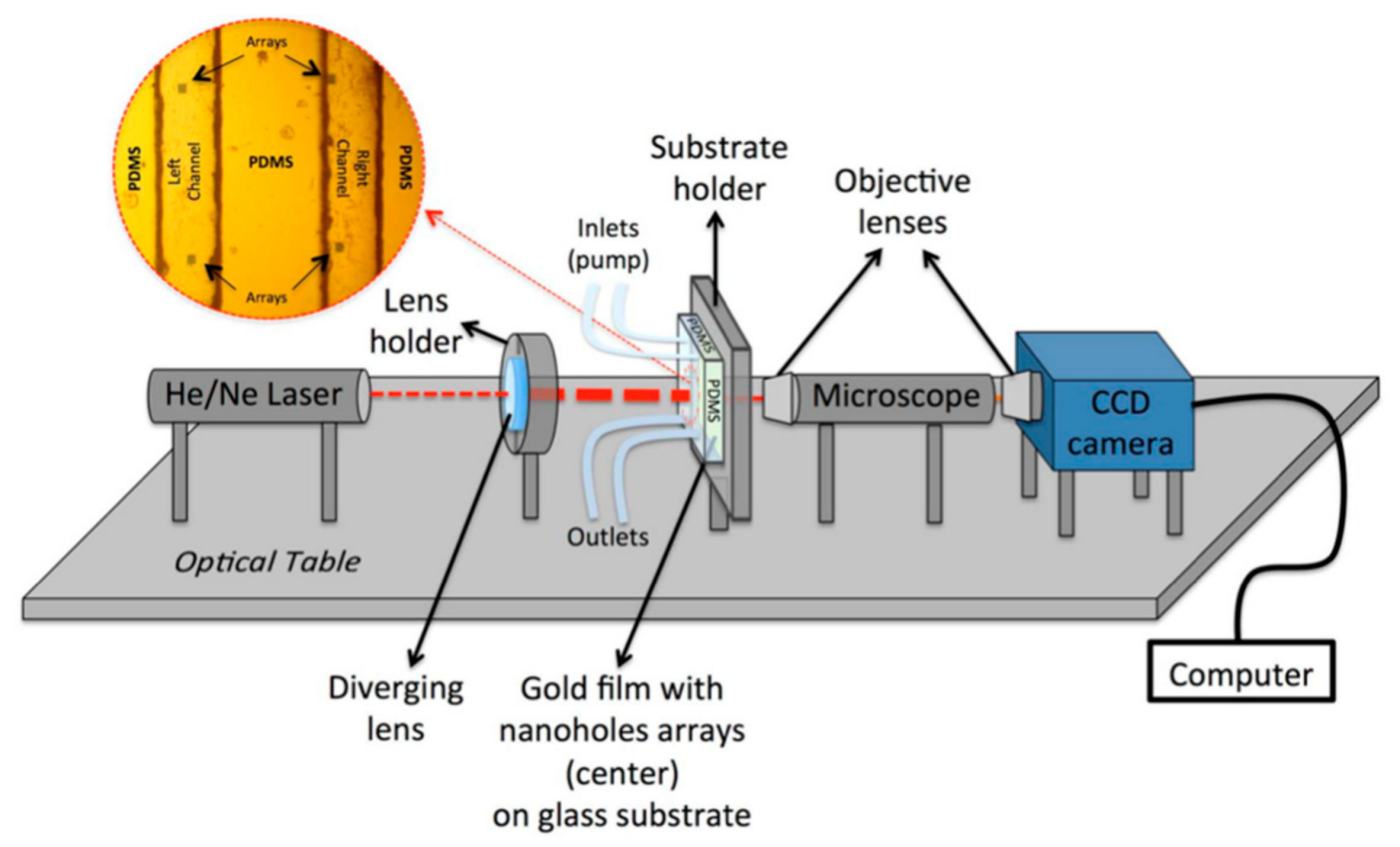
Experimental setup to monitor light intensity transmitted by nanohole array. Top insert: photographic image showing the arrays aligned with the microchannel manufactured on the PDMS piece. Adapted with permission from Ref. [31]. 2016, Monteiro, J.P.; Predabon, S.M.; Bonafé, E.G.; Mar-tins, A.F.; Brolo, A.G.; Radovanovic, E.; Girotto, E.M.

HER2 antigen is another crucial biomarker for breast cancer diagnosis traditionally studied by enzyme-linked immunosorbent assay (ELISA method). John P. Monteiro et al. developed a unique biosensor based on subwavelength nanohole arrays on gold thin film operating on a collinear transmission mode, ideal for miniaturized point-of-care device applications (Figure 17). Bioassay performance and prognosis were analyzed using a microfluidic system. The average sensitivity for refractive index variation (RI) was around 4146 intensity units/RIU, and the highest array sensitivity value was 4179 intensity units/RIU. Experimental results demonstrate that the system was able to monitor HER2 at a known concentration of 3 ng/mL, which shows credible potential for future bioassay diagnosis [1,3,25,31,32,33,34,35,36,37,38,39,40,41,42,43,44,45,46,47,48,49,50,51,52,53,54].

## 6. Conclusions and Future Prospective

Cancer diagnostics and treatment is a burning topic, as millions of people around the world are suffering, and a great many researchers and scientists are working tirelessly to find cures and preventions. There is an ever-growing demand for precise, fast, and reliable diagnostic tools, especially in the case of malignant tumors. Hyperbolic metamaterials have created an exciting development in biosensing and attracted much attention due to tunable dielectric constant or fill fraction allowing extraordinary sensitivity from visible to near-infrared (NIR) wavelength, making it easier to detect lower molecular weight biomolecules for label-free and multianalyte detection application.

SPR biosensors utilize a spectral interrogation scheme, whereas HMMs can detect viruses as low as 1 × 10^5^ M concentration via angular sensitivity. HMMs are an ideal next-generation label-free point-of-care device due to their precise measuring capability. They can easily detect target analytes in a foreign environment without false positives, which modern-day SPR techniques lack. Lab-on-a-chip or miniaturization is another area where HMMs excel. Recently researchers have been developing HMMs-based liquid biopsy devices. HMMs are an excellent replacement for semiqualitative labelled immunoassays, which are currently limited in terms of sensitivity and the number of analytes that can simultaneously be measured. MM-based biosensors could be a game changer in cancer studies. By analyzing low (pg/mL) concentration, valuable insight can be revealed, such as structural changes in biomarker molecules related to neurodegenerative diseases, and soon, continuous monitoring will allow physicians to develop tailored precision medicine. For example, customized biosensors can provide a molecular profile of patients.

Since hyperbolic metamaterials can detect target analytes in low concentrations, they can readily be used for microfluidic systems by addressing mass transport limitations and effectively increasing detection time.

HMMs can eliminate the need for expensive components such as valves and pumps by integrating metamaterials into capillary microfluidic systems that work on the surface tension principle. *Digital microfluidics* is a recent development that utilizes electrical forces to manipulate micro and even picolitre droplets without requiring pumps or valves. The demand for nanophotonic devices with precision sensitivity will continue to grow. However, transforming exciting scientific advances to day-to-day commercial applications requires new sensing platforms operating in a constantly changing biological environment [55,56,57,58,59,60].

## Figures and Tables

**Table 1:** Materials for specific spectral range and application [18].

Frequency	UV	Visible		NIR	MIR and THz
Materials	Ag/Al_2_O_3_	Ag/TiO_2_	Ag/Ti_3_O_5_	TiN/(Sc, Al) N	InGaAs/AlInAs
		Au/TiO_2_	Ag/laser-dye	Al: ZnO/ZnO	SiC/SiO_2_
		Ag/MgF_2_	TiN/(Sc, Al) N		Graphene
